# Association between Single Nucleotide Polymorphism of Vitamin D Receptor Gene FokI Polymorphism and Clinical Progress of Benign Prostatic Hyperplasia

**DOI:** 10.1155/2015/235895

**Published:** 2015-01-20

**Authors:** Li Ruan, Jian-guo Zhu, Cong Pan, Xing Hua, Dong-bo Yuan, Zheng-ming Li, Wei-de Zhong

**Affiliations:** ^1^Department of Urology, Guangzhou Red Cross Hospital, Medical College, Jinan University, Guangzhou 510220, China; ^2^Department of Urology, Guizhou Provincial People's Hospital, Guizhou 550002, China; ^3^Guizhou Provincial Key Laboratory of Computational Nano-Material Science, Guizhou Normal College, Guiyang 550018, China; ^4^Department of Pathology, Guangzhou Red Cross Hospital, Medical College, Jinan University, Guangzhou 510220, China; ^5^Department of Urology, Guangzhou First People's Hospital, Guangzhou Medical University, Guangzhou 510180, China

## Abstract

*Background.* The aim of the study was to investigate the association between single nucleotide polymorphism (SNP) of vitamin D receptor (VDR) gene and clinical progress of benign prostatic hyperplasia (BPH) in Chinese men.* Methods.* The DNA was extracted from blood of 200 BPH patients with operation (progression group) and 200 patients without operation (control group), respectively. The genotypes of VDR gene FokI SNP represented by “F/f” were identified by PCR-restriction fragment length polymorphism. The odds ratio (OR) of having progression of BPH for having the genotype were calculated.* Results*. Our date indicated that the f alleles of the VDR gene FokI SNP associated with the progression of BPH (*P* = 0.009).* Conclusion*. For the first time, our study demonstrated that VDR gene FokI SNP may be associated with the risk of BPH progress.

## 1. Introduction

Benign prostatic hyperplasia (BPH) is a commonly diagnosed disease in old men and it severely influences the quality of life of the patients. The status of clinical progression of BPH is vital because it provides guidance whether the treatment should be planned or not. Bladder weight and detrusor thickness, chronic prostatic inflammation, metabolic syndrome (MetS), and null or mild lower urinary tract symptoms have been used for predicting the progression of BPH [1–4], with limited accuracy. CD4^+^ and CD8^+^ T-lymphocytes cannot reliably predict the progression of BPH [5]. Up to now, no molecular markers for the progression of BPH have been fully established. Several molecular biomarkers, such as PSA, P25/26, TGFBRII, and uPSA, have been reported to have high potential to fill this void [6–8].

Studies have shown that vitamin D played an important role in the development of BPH through vitamin D receptor (VDR) [9]. Human VDR gene is located on the chromosomal locus 12q13-14, and it is considered the major gene that determines VDR concentration in cells. VDR is a member of the nuclear hormone receptor family which influences the function of genes that are involved in cell regulation, growth, and immunity [10]. More than 11 single nucleotide polymorphisms (SNPs) have been reported in VDR gene's coding and promoter regions by the genome-wide scans [11]. A few studies based on Asian cohorts as well as patients in western countries indicated that vitamin D receptor polymorphisms are associated with the incidence of prostate cancer, and vitamin D receptor may be a therapeutic target for BPH [12–14]. Moreover, FokI polymorphisms in promoter region have been frequently reported to be associated with nonspecific inflammation [15, 16]. As nonspecific inflammation, histological prostatitis (HP) may be an important factor that contributes to clinical progression of BPH [2]. The previous studies indicated that the SNPs of VDR gene FokI are associated with the incidence of BPH with HP [17].

Since BPH progression may lead to mutilating consequences, such as operation, it is clinically significant to develop biomarkers that can be used for predicting BPH progression. For the first time, we investigated the association between VDR FokI polymorphisms and progression of BPH using a Chinese cohort. The discovery of this study provided evidence of the association between VDR FokI polymorphisms and progression of BPH in Chinese population, and it is worthy of further validation in other ethnicities.

## 2. Materials and Methods

### 2.1. Materials

#### 2.1.1. The Experimental Subjects

This study has been approved by Guangzhou Red Cross Hospital Ethics Committee. 200 cases of BPH patients were collected from Department of Urology in Guangzhou Red Cross Hospital with surgical treatment from January 2010 to December 2012 (progression group: surgical procedureswere based on the guideline of BPH in China [2009 edition]) [8]. 200 cases of BPH patients were collected from outpatient Department of Urology in Guangzhou Red Cross Hospital without operation in the same period (control group). All patients were of the Han nationality in China. The patients in two groups were 70 to 80 years old, and there was no statistically significant difference in age between two groups (*P* > 0.05). The prostate volume, I-PSS (international prostate symptom score), PSA (prostate specific antigen), and *Q*_max⁡_ (maximum flow rate) were measured. All specimens of prostate were collected by transurethral prostate (TURP). All patients in the study have signed an informed consent for participation. All participants with TURP have signed the agreement of operation and transfusion. The pathological examination of the specimens was independently carried out by two pathologists in Guangzhou Red Cross Hospital.

#### 2.1.2. The Selection Criteria for Progression Group

(1) Patients have been using finasteride for one year or longer; (2) the PSA rose in watchful waiting or in the period of using drug (PSA > 4 ng/mL in follow-up with conformed BPH by biopsy) from January 2010 to December 2012; (3) the *Q*_max⁡_ decreased or have acute urine retention (AUR).

#### 2.1.3. The Selection Criteria for Control Group

(1) Patients have been using finasteride for one year or longer and (2) the patients did not have AUR, high value of PSA, change of prostate volume, or low value of *Q*_max⁡_ in period of taking finasteride from January 2010 to December 2012.

### 2.2. Methods

#### 2.2.1. DNA Extraction

Peripheral blood (2 mL) for DNA extraction was collected in tubes containing EDTA for both progression and control groups. DNA was isolated using the TIAN amp Genomic DNA Kit (Jingmei Corporation, Shenzhen, Guangdong, China).

#### 2.2.2. PCR Amplification

The VDR primers (Jingmei Corporation, Shenzhen, Guangdong, China) were designed by Genefisher based on the human VDR sequence (GeneBank Accession number: NC_000012.11) as 5′ ACTGACTCTGGCTCTGAC 3′ and 5′ CACCTTGCTTCTTCTCCC 3′. The amplification was performed in a volume of 50 *μ*L solution, including 2 *μ*L of DNA, 8 *μ*L of each primer, 2 *μ*L of dNTPs, 0.7 *μ*L of Taq DNA polymerase, and 1X of PCR buffer. The following steps were applied to amplification: (1) initial denaturation at 96°C for 8 minutes, (2) 40 cycles of 94°C for 45 seconds, (3) 52°C for 60 seconds, (4) 72°C for 30 seconds, and (5) a final elongation step at 72°C for 10 minutes.

#### 2.2.3. RFLP Analysis of SNP

**Figure 1:**
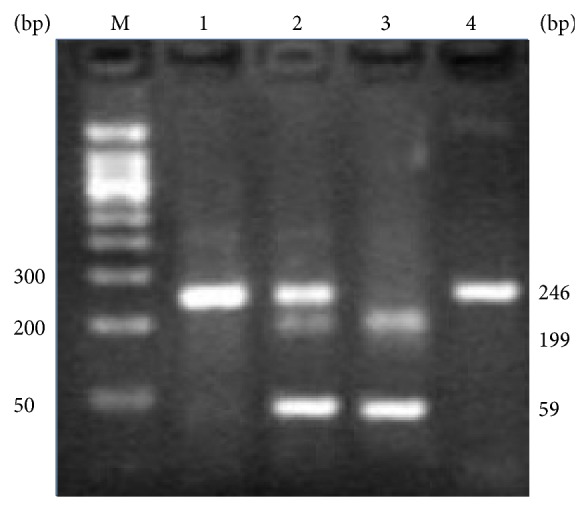
Restriction endonucleases with FokI for SNP “F/f.” M: marker; 1: genotype FF (246 bp); 2: genotype Ff (246, 199, and 59 bp 3 fragments); 3: genotype ff (199 bp and 59 bp 2 fragments); 4: original product of PCR amplification.

10 *μ*L of PCR products was digested with 0.5 *μ*L of FokI restriction endonuclease (Jingmei Corporation, Shenzhen, Guangdong, China), 1 *μ*L of BSA (Bovine Serum Albumin), 7.5 *μ*L of DNA, and 1 *μ*L of 10× buffer for 24 h at 37°C. The PCR products of digestion were analyzed by electrophoresis on 2% agarose gel stained with ethidium bromide. The fragments obtained for FokI SNP were 246 bp. The fragments for the “FF” genotype were 246, 199, and 59 bp, respectively. The fragments were 199 bp and 59 bp for the “Ff” genotype and the “ff” genotype, respectively (Figure 1).

### 2.3. Statistical Analysis

The main characteristics of the metric variables were expressed as mean and standard deviation (m ± St.Dev.). Student's *t*-test was used to compare continuous variables between two groups. Qualitative variables were summarized as percentages and associated with 95% confidence intervals (95% CI) calculated with an optimized binomial formula.

In the genetic analysis, we verified the Hardy-Weinberg equilibrium by applying the Chi-squared goodness-of-fit test performed using DeFinetti program (http://ihg.gsf.de/cgi-bin/hw/hwa1.pl). Adjusted odds ratio (OR) by age and their 95% confidence intervals (95% CI) were estimated using logistic regression analysis. Furthermore, logistic regression analysis was also applied to assess the effect of the SNP “F/f” polymorphisms on progression of BPH after controlling for covariates (variables with significant difference between progression group and control group).

Statistical analysis was performed using the SPSS software version 17 (SPSS Inc., Chicago, IL, USA). All *P* values were two-sided and were considered as significant when being less than 0.05. The *P* values were adjusted in compensation of multiple comparisons.

## 3. Results

Four hundred patients were included in our study, with 200 in the progression group and another 200 in the control group. Basic characteristics of patients in two groups are summarized in Table 1. Significantly larger prostate volumes have been observed in the progression group (41.51 ± 5.16) compared to the control group (40.33 ± 4.33) (*t*-statistics = −1.105; *P* value < 0.05). Furthermore, high levels of total PSA have both been observed in the progression group (3.59 ± 0.89) and the control group (3.27 ± 0.78) (*t*-statistics = 2.931; *P*-value = 0.22). For I-PSS, statistically significant difference was detected between two groups (progression group: 15.4 ± 2.5; control group: 15.3 ± 2.32; *t*-statistics = 7.135; *P* value = 0.018).

*Q*_max⁡_ was measured for patients in both groups. A total 95 patients in progression group were in hospital due to AUR; therefore, the urinary flow rate could not be measured for these 95 patients. As presented in Table 2, no significant difference was detected between two groups in terms of *Q*_max⁡_ (*t*-statistics = −9.127; *P* value = 0.11).

Genotype frequencies for all 400 subjects (200 patients in the progression group and 200 patients in the control group) were shown in Table 3. There was no significant difference in genotype frequencies between actual and expected (*P* = 0.129 for the progression group; *P* = 0.396 for the control group). The distribution of SNP was in agreement with Hardy-Weinberg equilibrium for both groups. Genotype and allele frequencies of FokI in the progression group and the control group were shown in Table 4. Statistically significant difference has been identified in both genotype and allele frequencies between two groups (genotype: *P* = 0.042; allele: *P* = 0.009).

The association between the susceptibility of progression of prostate inheritance and VDR FokI polymorphisms was tested using logistic regression analysis. The forward likelihood ratio (LR) method was applied to exclude the effects of confounders (such as age, prostate volume, I-PSS, PSA, and *Q*_max⁡_). The forward Wald analysis revealed that the intercept was significant in univariate linear regression model. The results showed that the VDR FokI genotype “ff” is substantially associated with progression of BPH (Table 5).

## 4. Discussion

The association between VDR FokI polymorphisms and BPH progression has not been previously studied yet. In the current study, the association between SNPs of VDR gene FokI and progression of BPH was evaluated by comparing a group of patients with BPH progression to a group of control patients. The differences in the genotype of VDR FokI SNP were identified between the progression group and the control group (Table 3). The variants in the current study are novel and have not been reported in recent genome-wide association studies. We identified the risk allele for SNP FokI. The distributions of the three genotypes of VDR FokI SNPs were significantly different between progression group and control group, indicating that VDR FokI SNPs may be a risk factor for BPH progression and subject with heterozygote of “ff” is likely to develop BPH progression (Table 5: *P* = 0.009).

Two patient groups were similar in the majority of characteristics (see Table 1). Regarding anthropometrics, the progression group has significantly larger volume of prostate (*P* = 0.035) and higher level of I-PSS (*P* = 0.018) than the control group. Neither age nor PSA was significantly different between the progression group and control groups (Table 1: *P* > 0.05). PSA may be influenced by drug such finasteride or prostatitis. The *Q*_max⁡_ was not significantly different between two groups either (Table 2: *P* > 0.05). *Q*_max⁡_ may be influenced by the application of drug such as tamsulosin. According to our result, we thought *Q*_max⁡_ and PSA appear not to be good indicators for progression of BPH.

The association between age and progression of BPH has been previously identified [18]. The associations between *Q*_max⁡_ and PSA and progression of BPH have also been reported in the previous studies [19, 20]. Catheterization, DRE (digital rectal examination), and biopsy of prostate could affect the level of PSA. On the other hand, *Q*_max⁡_ may be influenced by the application of drug. *Q*_max⁡_ and PSA appear not to be good indicators for progression of BPH. In the current study, we found that the control group has smaller prostate volume and lower level of I-PSS than the progression group (*P* < 0.05), which agreed with the results from other studies [2, 21].

The current study has two major limitations. (1) Replication is useful in genetic studies; however, no replication is available in the study due to the absence of the SNPs from the recent genome-wide association studies of BPH progress. (2) Only Chinese patients were investigated in the study. Nevertheless, the significant data from the current study strongly suggested that VDR FokI SNPs are associated with BPH progression. Well-designed observational studies (e.g., matched case-control studies) including multiple ethnicities may be useful for further validation.

## 5. Conclusion

The assessment of association between BPH and VDR genes BsmI and TaqI SNPs has not been reported in the literatures. Thus, this is the first study that was conducted to investigate the link between VDR gene FokI SNP and BPH progress. The main findings of the study are summarized as follows: (i) *Q*_max⁡_ and PSA are not good indicators for progression of BPH; (ii) VDR gene FokI SNPs may be a molecular markers which is associated with progression of BPH; (iii) genotype “ff” is likely to develop BPH progression.

## Figures and Tables

**Table 1:** Anthropometric and biochemical characteristics by groups.

Variable	Progress group (*n* = 200)	Control group (*n* = 200)	*P* value
Age	73.9 ± 2.7	73.56 ± 2.68	0.13
Volume of prostate (mL)	41.51 ± 5.16	40.33 ± 4.33	0.035
Total PSA (ng/mL)	3.59 ± 0.89^*^	3.27 ± 0.78^*^	0.22
I-PSS	15.4 ± 2.5	13.3 ± 2.32	0.018

^*^The decrease of PSA levels was 50 percent for the patients treated with finasteride.

**Table 2:** The *Q*_max⁡_ of two groups.

Variable	Progress group (*n* = 105)	Control group (*n* = 200)	*P* value
*Q*_max⁡_ (mL/s)	9.47 ± 2.02	10.67 ± 1.48	0.11

**Table 3:** The equilibrium test of Hardy-Weinberg in two groups for FokI genotype.

Group	*n*	Genotype N						*χ* ^2^	*P*
		FF		Ff		ff	
		Obs	Exp	Obs	Exp	Obs	Exp
Progress	200	51	40.95	79	99.1	70	59.95	4.103	0.129
Control	200	66	59.41	86	99.19	48	41.4	1.854	0.396

**Table 4:** Genotype and allele frequencies of FokI in two groups.

Group	*n*	Genotype N (%)			Allele N (%)	
		FF	Ff	ff	F	f
Progress	200	51 (25.5%)	79 (39.5%)	70 (35%)	181 (45.25%)	219 (54.75%)
Control	200	66 (33%)	86 (43%)	48 (24%)	218 (54.5%)	182 (45.5%)
		*χ* ^2^ = 6.322 *P* = 0.042			*χ* ^2^ = 6.845 *P* = 0.009	

**Table 5:** Association analysis for VDR FokI SNP polymorphisms and BPH progress.

	Coefficient	Wald	OR	95% CI	*P*
Constant term	−1.404	12.447	0.246		<0.001
ff genotype	0.764	6.740	2.146	(1.206~3.821)	0.009
Ff genotype	0.336	1.477	1.399	(0.814~2.403)	0.224
